# Social interactions in isolated, confined, and extreme environments: A study of Antarctic winter teams using wearable sensors

**DOI:** 10.1073/pnas.2533420123

**Published:** 2026-05-26

**Authors:** Andrea Cantisani, Jan B. Schmutz, Pedro Marques-Quinteiro, Lorenzo Dall’Amico, Ciro Cattuto, Mirko Antino, Walter J. Eppich, Katharina Stegmayer, Sebastian Walther

**Affiliations:** ^a^Translational Research Center, University Hospital of Psychiatry and Psychotherapy, Bern 3000, Switzerland; ^b^Sanatorium Kilchberg, Private Clinic for Psychiatry and Psychotherapy, Kilchberg 8802, Switzerland; ^c^Department of Psychology, University of Zurich, Zurich 8050, Switzerland; ^d^Intrepid Lab, School of Economic Sciences and Organizations, Lusófona University, Lisboa 1749-024, Portugal; ^e^Centre for Transdisciplinary Development Studies, Universidade de Trás-os Montes e Alto Douro, Vila Real 5000-801, Portugal; ^f^Institute for Scientific Interchange Foundation, Torino 10123, Italy; ^g^Department of Methodology for Behavioral Sciences, Universidad Complutense de Madrid, Madrid 28040, Spain; ^h^Faculty of Medicine, Dentistry, and Health Sciences, University of Melbourne, Melbourne, VIC 3010, Australia; ^i^Department of Psychiatry, Psychosomatics, and Psychotherapy, Center of Mental Health, University Hospital of Würzburg, Würzburg 97080, Germany

**Keywords:** team dynamics, social isolation, ICE environments, space analogs, social networks

## Abstract

Long-duration space missions expose crews to extreme psychological and social stressors due to prolonged isolation and confinement. To examine how such conditions impact individual and team functioning, we studied a 10-mo Antarctic overwintering mission at Concordia Station, an analog for spaceflight, using self-report measures and wearable proximity sensors. Twelve crew members were assessed at four time-points. Results revealed a progressive increase in feelings of loneliness and conflict, while cohesion and individual performance declined. Close-range interactions were positively associated with conflicts and paranoid thoughts and negatively related with individual performance, suggesting that more frequent contact did not equate to social support. Social interactions became increasingly clustered within national groups, highlighting the risk of social fragmentation and crew polarization. The use of wearable sensors proved feasible for long-term use in extreme environments. These findings underscore the importance of monitoring psychosocial functioning during extended missions and suggest that close confinement, rather than isolation alone, may trigger interpersonal strain.

## Significance

Teams living and working in isolated, confined, and extreme (ICE) environments—such as Antarctic stations or future missions to the Moon and Mars—face unique psychological and social challenges. Yet longitudinal, high-resolution data on how social interactions and team functioning evolve during prolonged real-world isolation remain scarce. Using wearable proximity sensors and repeated psychological assessments during a 10-mo Antarctic overwintering mission, we provide a detailed account of how social contact, cohesion, conflict, and feelings of loneliness and mistrust change over time. Our findings reveal emerging interpersonal strain, subgroup formation, and increased suspiciousness despite close physical proximity. These results highlight critical psychosocial risks for teams in ICE environments and the value of continuous, objective monitoring of crew dynamics.

Human space exploration is on the brink of a new era, with space agencies and private enterprises planning long-duration missions to the Moon, asteroids, and eventually Mars (1). These exploratory missions will push the boundaries of human endurance as crews face unprecedented levels of isolation, limited rescue possibilities, and extreme psychological and physiological challenges (2). Lasting months or even years, such missions will require small teams to operate in confined and isolated spaces, under hazardous conditions and high autonomy requirements, with significant communication delays with Earth (3).

Space, as an extreme environment, presents persistent physical dangers such as increased radiation exposure and microgravity, which lead to muscle atrophy, the disruption of the vestibular systems, decreased bone density, and the risk of genetic mutations leading to cancer (4). In addition, the constant threat of equipment failure endangers crew safety and adds to psychological strain. Beyond these physical challenges, crews will also face psychological stressors from confinement and isolation, which can negatively impact mental health (5). Due to the intense physiological and psychological demands of isolated, confined, and extreme (ICE) environments, healthy interpersonal dynamics and team cohesion are vital for mission success (6, 7). High crew interdependence means that teamwork breakdowns or conflict can pose serious risks to mission outcomes and crew survival (1). Teams must maintain collaboration and mutual support under stress. Space agencies prioritize understanding these risks, and terrestrial ICE analogs (e.g., Antarctic research stations) provide valuable opportunities to study team functioning in space-like conditions (8, 9).

Antarctic overwintering crews have become a popular focus for research due to their similarity to long-duration space missions. These analog environments share key characteristics with space missions, such as heavy reliance on technology for life support, hostile external conditions, limited rescue options, communication delays, confinement, and multicultural teams working in small, isolated settings (8). Specific space analog stations, like NASA’s HERA in Houston (10), HI-SEAS in Hawaii (11), and LunAres in Poland (12), provide realistic simulations of missions to the Moon or Mars. However, these simulations are often limited by their shorter duration (typically 2 wk to 3 mo) and the fact that they remain accessible in case of emergencies, thus lacking the full psychological stressors of an actual space mission. For this reason, researchers have turned to Antarctic overwintering crews to study the effects of prolonged isolation and confinement. The harsh conditions at Antarctica’s Concordia Station, where this study was conducted, are particularly relevant. Its extreme remoteness, even surpassing that of the International Space Station (ISS), necessitates exceptional preparedness for self-sufficiency during emergencies and places significant stress on the operating crews (13).

Despite growing interest in teams operating in ICE environments (14), the literature on the psychological impact of long-duration missions remains limited in two key areas. First, there is a lack of comprehensive evidence clarifying how prolonged isolation affects individual psychological outcomes, and more critically, team dynamics, such as interactions and cohesion. Research on Antarctic overwintering crews has investigated individual variables, such as mood and sleep, consistently showing a decline in these measures over the course of the missions (15–18). However, empirical studies addressing specific psychological variables, such as perceived social isolation and feelings of mistrust, as well as team-level changes, particularly in team climate and cohesion, are far less common. In addition, existing studies on team cohesion in space analog missions have been limited to shorter durations, typically no longer than 4 mo (19, 20).

Second, there is a significant gap in our ability to reliably monitor team interactions over extended periods. Traditional survey-based methods can become burdensome for crews and are often impractical for capturing high-resolution data (e.g., multiple daily measurements), which impedes a reliable and granular view of a dynamic phenomenon. Given that team processes are inherently dynamic and evolve over time (21, 22), continuous monitoring is essential to enable timely interventions. Further, in the analysis of human interactions, particularly in ICE contexts, it is essential to combine nonintrusive observational methods with high ecological validity, such as geolocated sensing technologies that passively capture spatiotemporal patterns of interpersonal contact. This integration is critical because the extreme isolation inherent in ICE settings can distort individuals’ perceptions of the frequency and quality of their social interactions. While self-reports offer valuable insights into subjective experience, triangulating these perceptions with objective behavioral metrics reduces potential biases.

We aim to address these gaps by studying an overwintering crew at Concordia Station in Antarctica, using a combination of self-report measures and wearable proximity sensors. We deployed the SocioPatterns proximity sensors, a nonintrusive technology that has been used to quantify human close-range interactions, enabling the application of network science methods to investigate team dynamics (23–25). Proximity sensors have been deployed in various real-world settings, including challenging environments such as rural villages in sub-Saharan Africa (26, 27). Research has demonstrated that face-to-face interaction networks, measured with proximity sensors, can be linked to mental health, including depressive symptoms and feelings of isolation (28, 29), and have been associated with creativity in teams (30). Focusing on ICE environments, initial studies have started using wearable sensors to monitor interactions in small teams during 4-mo space analog missions (19, 20, 31) and investigated social networks for isolated crews in comparison with nonisolated crews (32).

Our first objective is to investigate how feelings of isolation and paranoia, as well as team cohesion, conflict, and perceived individual performance, evolve over the course of a 10-mo mission. A growing body of research shows that perceived social isolation is linked to adverse physical and mental health outcomes (33). Paradoxically, the need for social connection may heighten implicit hypervigilance to social threats (34, 35). Such bias can foster mistrust and suspiciousness (36, 37), which can be understood as attenuated forms of paranoid thinking, defined as unfounded or exaggerated beliefs that others intend harm and are relatively common even in nonclinical populations (38, 39). Importantly, paranoid-like ideation is associated with occupational stress and disrupted sleep (38, 40). These stressors are amplified in ICE environments, interact in detrimental ways, and may pose a substantial risk to team functioning.

On a team level, group cohesion refers to the motivational forces that drive members to remain united in pursuit of their objectives (41). When cohesion is strong, a team is motivated to perform well and better able to coordinate activities for successful performance (41). This effect may be even more pronounced for teams in confined environments, due to the forced interactions (42). In contrast, team conflict, defined as perceived incompatibilities or differences among group members (43), represents a significant risk factor for both individual mental well-being and team functioning (44). Conducting long-term studies in ICE environments is time-intensive, and sample sizes are inherently small. Therefore, this research contributes to the body of knowledge by replicating and extending findings on the psychological experiences of crews facing long-duration missions (18).

Our second objective is to evaluate the feasibility and utility of wearable proximity sensors as a long-term monitoring tool for team interactions in ICE environments. By deploying this technology during an overwintering mission at Concordia Station, we test whether proximity-based interaction networks can be reliably captured over extended periods and linked to psychological experiences. This initial application, under conditions analogous to long-duration space missions, may establish a foundation for the broader use of this technology in space research and other extreme settings.

## Results

### Questionnaires.

**Fig. 1.**
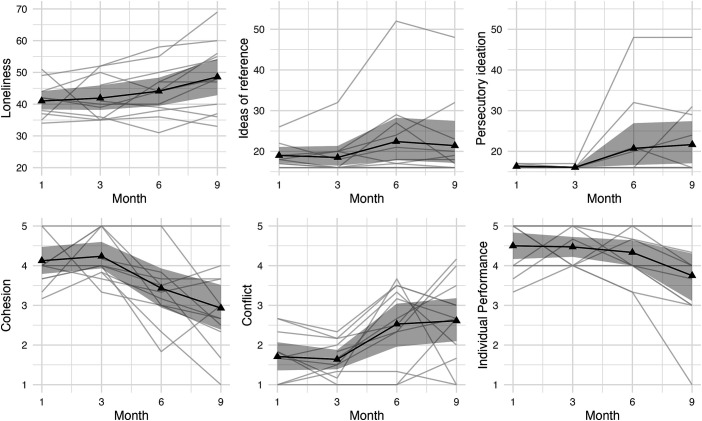
Evolution of psychological and team-related variables over a 9-mo period. Longitudinal trajectories for the six outcomes across four time points (months 1, 3, 6, 9). The solid black lines represent group means, shaded gray areas depict 95% bootstrap CI, and light gray lines indicate individual participant trajectories. Sample size varies between 9 and 12 due to missing data. Scales range: Loneliness, 20 to 80; Ideas of Reference and Persecutory Ideation, 16 to 80; Cohesion, Conflict, and Individual Performance, 1 to 5.

Fig. 1 illustrates the results for loneliness, the two dimensions of paranoid thoughts (i.e., ideas of reference, persecutory ideation), cohesion, conflict, and individual performance across the four measurement points.

We conducted repeated-measures ANOVAs, including only participants with complete data across all time points. The analysis revealed a significant main effect of time on loneliness, *F*(3, 24) = 3.41, *P* = 0.03 (*N* = 9), indicating an increase in loneliness over the course of the mission. To further examine change over time and to incorporate all available observations, we estimated a latent growth model. The model indicated a positive slope of time (*estimate* = 2.42, *P* = 0.01). Bootstrap CI (2,000 resampling) supported this effect (95% CI [0.85, 4.01]).

No significant main effect of time was found for ideas of reference or persecutory ideation. However, visual inspection of the data suggested a notable increase in both dimensions between Month 3 and Month 6. A post hoc paired-samples *t* test confirmed a significant increase in ideas of reference, from Month 3 (*M* = 18.50, *SD* = 4.60) to Month 6 (*M* = 22.42, *SD* = 10.39), *t*(11) = –2.11, *P* = 0.03 (*N* = 12). No significant change was observed for persecutory ideation in the same interval. The latent growth model indicated a positive slope of time for ideas of reference (*estimate* = 1.35, *P* = 0.03), as well as for persecutory ideation (*estimate* = 2.35, *P* = 0.01). Bootstrap CI (2,000 resampling) supported both effects (ideas of reference; 95% CI [0.07, 3.11] and persecutory ideation; 95% CI [0.33, 4.52]).

One participant reported unusually high scores (exceeding 40) on both paranoid thoughts subscales, corresponding to severe levels of paranoid ideation according to established thresholds (45). Given the extreme nature of these values and the unlikelihood that an individual experiencing such levels of paranoia could function effectively in the highly interdependent, confined team environment, we conducted a sensitivity analysis excluding this individual. The increase in ideas of reference between Month 3 and Month 6 remained statistically significant.

For team-level outcomes (based on *N* = 10 complete cases), results showed a decline in cohesion over time, *F*(3, 27) = 7.41, *P* < 0.001, and in individual performance, *F*(3, 27) = 3.56, *P* = 0.03. Team conflict increased over time, *F*(3, 27) = 4.82, *P* = 0.01. The latent growth models show coherent results with a negative slope of time for cohesion (*estimate* = −0.42, *P* = 0.01, 95% CI [−0.59, −0.27]), individual performance (*estimate* = −0.24, *P* = 0.01, 95% CI [−0.48, −0.07]), and a positive slope for conflict (*estimate* = 0.35, *P* = 0.01, 95% CI [0.19, 0.53]).

We refer the reader to *SI Appendix*, Table S1 for the full correlation matrix, including means (M) and SD of all study variables assessed at the four time points through questionnaires.

### Proximity Sensors.

**Fig. 2.**
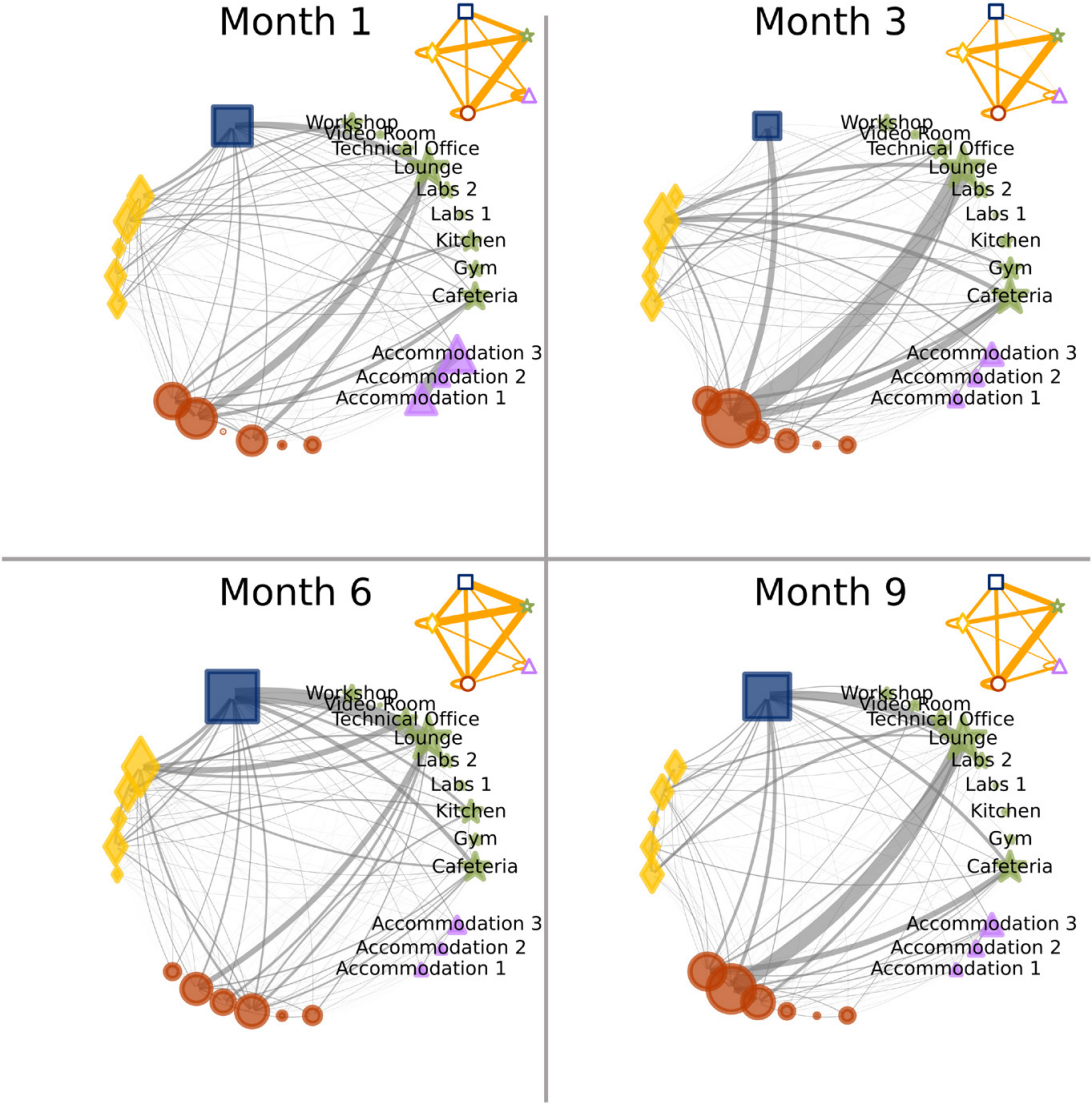
Aggregated contact networks measured with wearable proximity sensors across the four deployment periods (Months 1, 3, 6, and 9). Each node represents a sensor. Red circles and orange diamonds correspond to members of the two nationality groups; the blue square represents the ESA medical doctor; green stars correspond to stationary sensors placed in shared rooms; and purple triangles represent stationary sensors located near the accommodation areas. Nodes are ordered identically across all panels to enable comparison over time. For the two crew members who were evacuated during the mission, their positions were retained and the incoming crew members were plotted in the same locations. Individual identifiers and nationalities were omitted to preserve anonymity. Edge thickness is proportional to the cumulative duration of face-to-face proximity between a pair of sensors during the respective measurement period. Each *Inset* shows an aggregated network in which nodes are grouped by category (nationality groups, ESA medical doctor, accommodation areas, and shared rooms); edge thickness represents the total interaction time summed over all pairs of nodes belonging to the connecting groups.

The wearable proximity sensors performed reliably under the extreme environmental conditions at Concordia Station in Antarctica. The sensors consistently captured high-resolution data on face-to-face interactions throughout the study. Fig. 2 illustrates the aggregated proximity networks, measured using proximity sensors at four time points. The nodes (i.e., entities) represent individuals [red dots and orange diamonds represent the two nationality groups, the blue square represents the European Space Agency (ESA) medical doctor] and rooms (green stars are shared rooms, while purple triangles are spaces near the accommodation areas). The edges (i.e., links) represent the connections between entities. The thickness of each edge reflects the cumulative interaction time between nodes. For instance, the ESA medical doctor shows notably increased time spent in the lounge during Months 6 and 9 compared with Months 1 and 3. The insets display aggregated group-level interaction patterns between the two nationality groups, the ESA medical doctor, and the shared spaces.

**Fig. 3.**
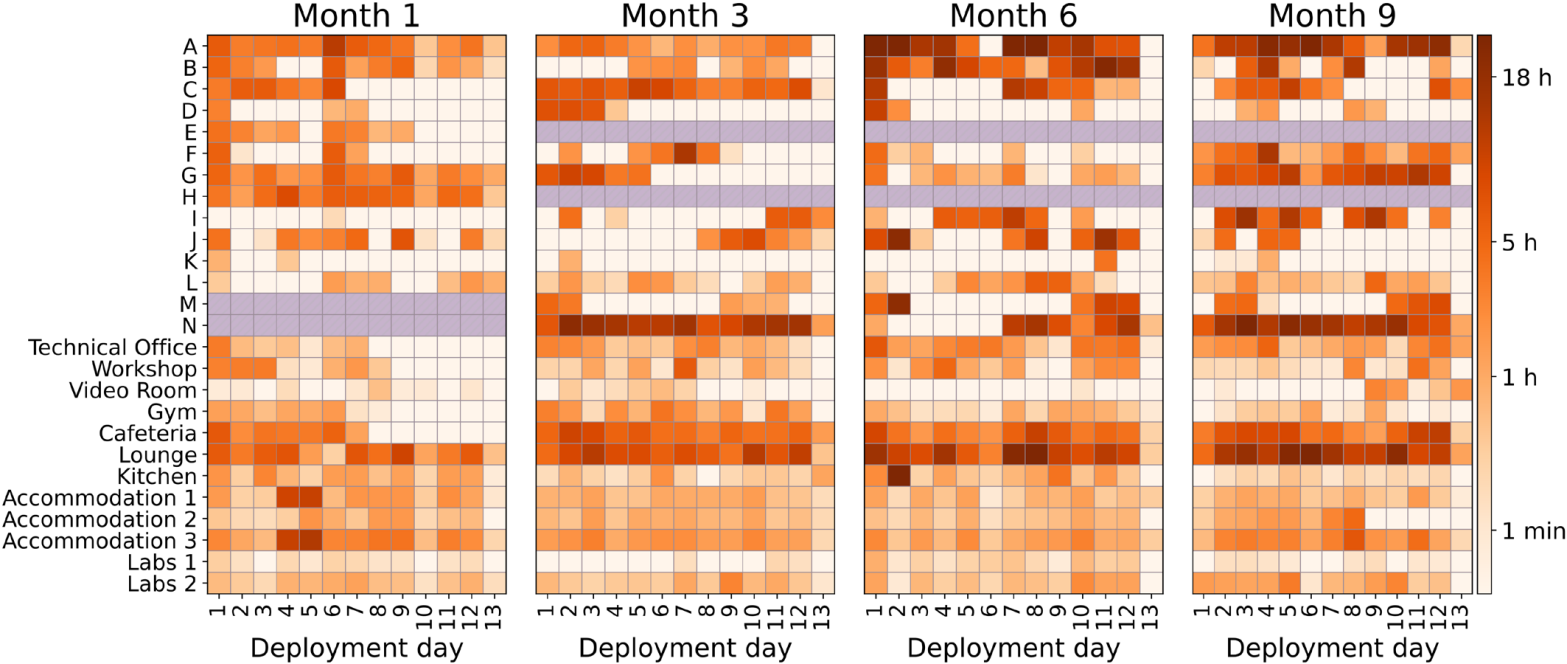
Color-coded strength centrality per day, subdivided by the four deployment periods for people (marked with letters) and rooms. The darker the color, the more interactions happened on that specific day. Two team members (E and H) left the mission after the first data collection due to medical reasons and were replaced by subjects M and N; therefore, cells with diagonal lines mark days when individuals were not present, and no proximity data are available.

Fig. 3 illustrates the strength centrality metric computed for each participant (labeled A–N) and specific rooms on a given day during the deployment period. Darker colors represent higher strength, while lighter colors indicate lower strength. As expected, common areas such as the cafeteria and lounge, where crew members typically gather to eat and relax, show the highest strength, in agreement with Fig. 2. This alignment with expected patterns of social interaction provides additional evidence about the validity of the sensor-based interaction data.

**Fig. 4.**
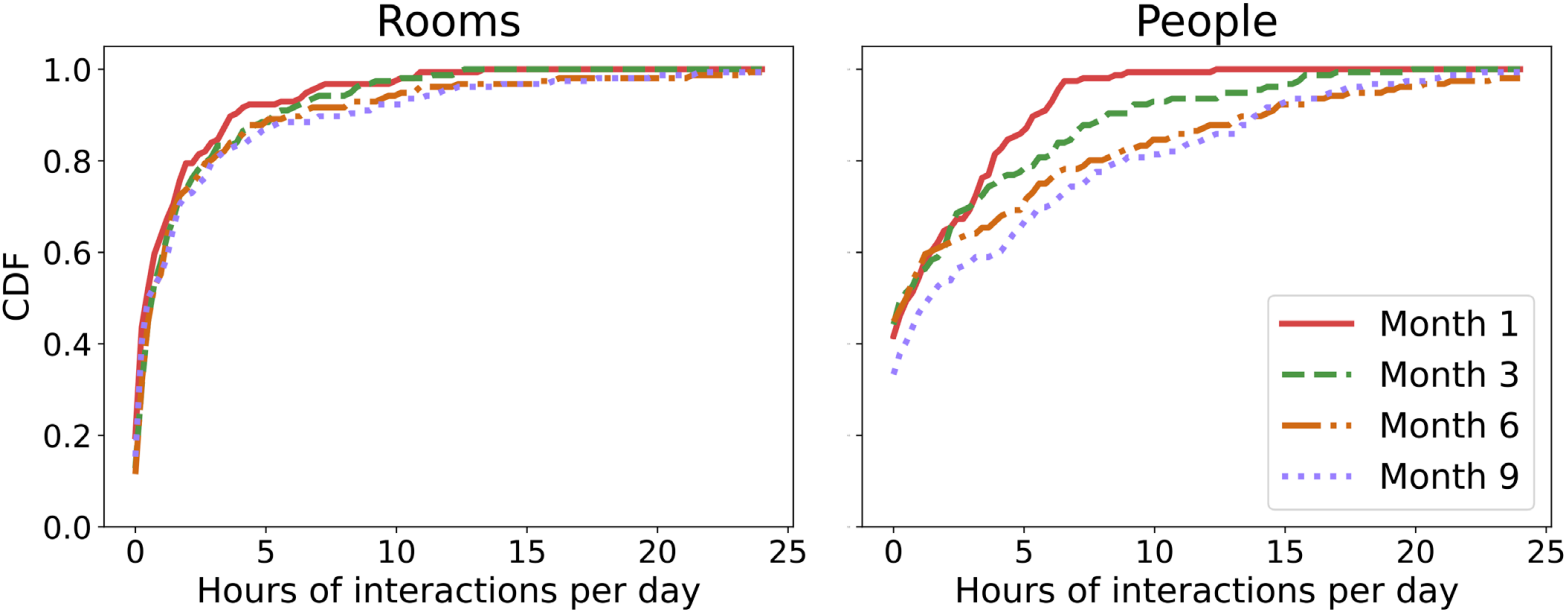
CDF of daily strength centrality. Each line represents one of the four deployments, represented with different colors and styles. The left plot is for room sensors, while the right one is for human subjects.

Fig. 4 illustrates the cumulative density function (CDF) of daily strength centrality across the four deployment periods, separately for stationary room sensors (*Left*) and wearable person sensors (*Right*). The CDF represents the cumulative proportion of sensor–subject day pairs with up to a given number of interaction hours. For example, a CDF value of 0.8 at the 5-h mark indicates that 80% of sensor–subject days recorded 5 h or fewer of interaction. This figure complements Fig. 3 by offering a distributional summary of interaction time across deployments.

For sensors worn by human subjects (right), the CDF shifts notably from Month 1 to Month 9 (Kolmogorov–Smirnov test between the duration distributions of the first and last deployment, *P* = 10^−4^), indicating a decrease in daily interaction duration among participants over time. In contrast, the stationary sensors in rooms (left) have relatively stable distributions, suggesting consistent room usage patterns throughout the mission.

The value of the CDF at zero represents the proportion of sensor–subject days with no recorded interactions. This proportion is higher for wearable person sensors (~40% of sensor-day pairs with zero contacts) than for stationary room sensors (~10%). Because stationary sensors are not affected by participant adherence and showed consistently low rates of zero-interaction days, this discrepancy suggests that part of the zero values in the person-sensor data reflects lapses in wearing compliance rather than technical malfunction alone.

At the same time, the data do not allow us to distinguish with certainty between true absence of interaction and temporary nonwear. Field observations from the ESA medical doctor indicated no evidence of major generalized noncompliance but suggested that some instances of nonwear were deliberate and occasionally coincided with periods of voluntary physical or social withdrawal. This observation is in line with the study design that explicitly allowed crew members to withdraw the study at any moment, even temporarily. Accordingly, zero-interaction days in the wearable data likely reflect a combination of true low interaction and intermittent nonwear.

**Fig. 5.**
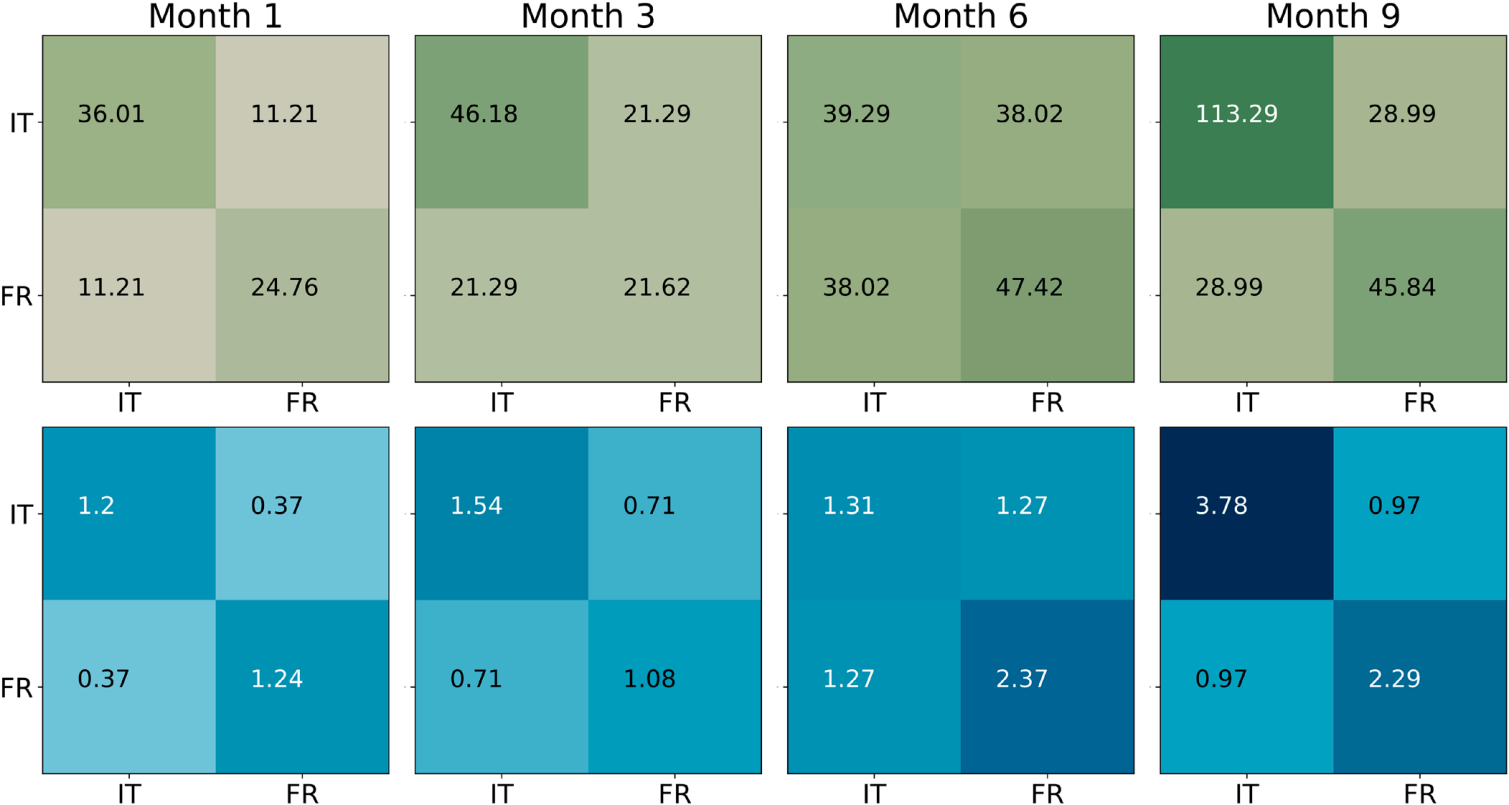
Contact matrices between national groups. The *Upper* row represents raw contacts in hours (*Top*) between Italian (IT) and French (FR) participants. The *Bottom* row shows the same quantities rescaled by the total number of possible interacting pairs.

Finally, Fig. 5 shows a high assortativity of the contact patterns with respect to nationality, meaning that crew members preferentially interacted with others who shared their own nationality. This tendency to form same-group connections was especially pronounced toward the end of the mission in Month 9.

### Relationship between Questionnaire and Proximity Sensor Data.

**Fig. 6.**
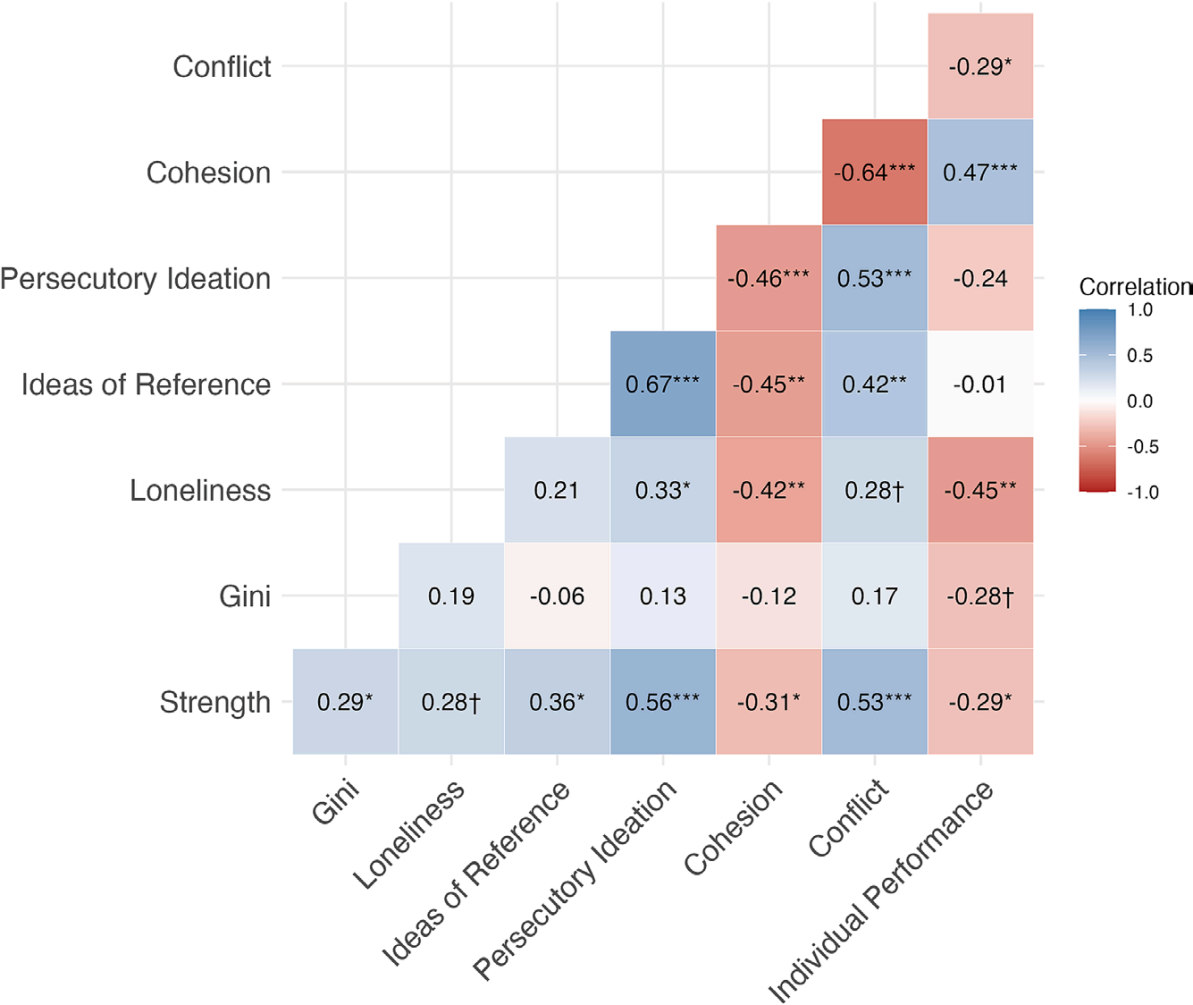
Correlation heatmap displaying correlations among study variables. Correlations are based on person-mean centered variables and reflect within-person associations. Numbers represent Spearman correlation coefficients (two-tailed), color-coded from red (negative) to blue (positive) to indicate the strength and direction of association. ***P < 0.001, **P < 0.01, *P < 0.05; † denote marginal significance (*P* < 0.10); *N* = 48.

Fig. 6 reports the Spearman correlation matrix between proximity-based measures (i.e., strength centrality and Gini coefficient) and self-reported variables across the four measurement points (*N* = 48). The Gini coefficient only showed a trend-level association with individual performance (*P* = 0.06), indicating that inequality in physical contact distribution was mostly unrelated to psychological or social outcomes in this context.

In contrast, strength centrality, reflecting the overall amount of physical proximity, showed positive correlations with ideas of reference, persecutory ideation, and conflict, suggesting that individuals with more frequent contact also reported higher levels of paranoid ideation and team tension. Further, strength centrality was negatively related with cohesion and individual performance. While not statistically significant at the 0.05 level, strength showed a trend-level, positive correlation with loneliness (*P* = 0.06).

## Discussion

This study examined how individual psychological variables, in particular perceived social isolation, paranoid thoughts, and perceived individual performance, and team-level dynamics, such as cohesion and conflict, evolved over the course of a 10-mo overwintering mission at Concordia Station. Combining self-report data with high-resolution proximity sensor data, we sought to understand psychosocial functioning in one of the most ecologically valid analogs for long-duration spaceflight (42). Our findings advance the understanding of psychological and interpersonal adaptation in ICE environments, while demonstrating the potential of wearable sensors for continuous, unobtrusive monitoring of team dynamics in these settings.

Overall, we observed a progressive deterioration in both individual psychological outcome and team dynamics (i.e., an increase in loneliness and conflict, and a decrease in cohesion and performance). Loneliness and paranoid thoughts increased over time, team cohesion declined, interpersonal conflict intensified, and perceptions of individual performance diminished. These patterns are broadly consistent with prior findings from space analog missions and polar expeditions (42, 46) and underscore the psychological toll of extended confinement and isolation under extreme environmental conditions.

Previous research in polar stations and space analog environments has documented increases in negative affect, cognitive fatigue, and sleep disturbances over time (46). Consistent with these studies, we found that indicators of individual well-being and positive team functioning declined as the mission progressed. Sandal et al. (47) reported similar trends during a prior mission at Concordia, with declines in positive affect and in the use of both problem-solving and avoidance-based coping strategies over time. Our findings support the notion that under chronic isolation, motivational and emotional resources become depleted, potentially impairing both psychological resilience and social cohesion.

Loneliness scores progressively increased and reached levels comparable to moderate social isolation in the general population (48), a striking observation given the structured team context and limited access to external communication. While limited Internet connectivity may have helped buffer more severe isolation, loneliness was nonetheless negatively associated with team cohesion, suggesting that feelings of disconnection may erode group bonds. Loneliness was associated with increases in conflict and paranoid ideation and negatively associated with perceived individual performance. This aligns with prior research suggesting that loneliness heightens social vigilance and mistrust, potentially fueling interpersonal tension and reducing performance (49, 50).

Paranoid thoughts also increased over time, particularly in the form of ideas of reference, milder forms of suspiciousness involving the perception that others are commenting on or observing oneself. These subclinical experiences are relatively common in the general population (39, 51) but their escalation in this context is noteworthy. Applying severity thresholds from a revised version of the Green et al. Paranoid Thought Scales (GPTS; 45), participants reported elevated levels of mistrust by mid-mission, despite having undergone rigorous psychological screening. This highlights the potential for even psychologically resilient individuals to develop distorted social perceptions under extreme conditions. Many of the suspected contributors to paranoid ideation are present in ICE environments and were observed in our data (e.g., loneliness, sleep disturbance, and safety seeking behaviors such as social avoidance). Thus, the findings in ICE environments support the general model of paranoid ideation (52).

In line with prior reports (53, 54), we observed an increase in team conflict over time. These interpersonal tensions were strongly negatively correlated with cohesion and individual performance and positively correlated with loneliness and paranoid thoughts. Given the correlational nature of our data, we can only speculate about the directionality of these relationships. Conflict may be both a consequence and a contributor to psychological strain: As team cohesion deteriorates and individuals begin to feel disconnected or mistrustful, the likelihood of interpersonal friction may increase. In turn, recurring conflict could reinforce negative social perceptions, heighten feelings of isolation, and diminish individuals’ sense of competence and motivation. The observed positive associations between conflict, loneliness, and paranoid thoughts may point to a possible feedback loop, which might be explained through cognitive-behavioral models of paranoia and loneliness (49, 55). These models suggest that perceived social disconnection heightens sensitivity to social threat and fosters negative interpretations of others’ behavior. In this view, feelings of loneliness and mistrust may lead individuals to withdraw or respond defensively, prompting negative reactions from others that reinforce the original fears. In our study, the positive associations between loneliness, paranoid thoughts, and conflict are consistent with such a self-reinforcing loop. In the resource-limited and high-stakes context of an ICE environment, these spirals may be especially difficult to interrupt, gradually undermining both individual well-being and team functioning. In extreme environments where effective collaboration is mission-critical, even subtle interpersonal tensions may escalate quickly, underscoring the need for early detection and proactive strategies to manage team conflict.

While interpersonal interactions in this confined environment, as captured by wearable sensors, tended to increase across time, these increases were not associated with improved well-being or team functioning. On the contrary, higher levels of proximity-based interaction were positively correlated with conflict and paranoid ideation—variables typically considered detrimental to psychological and social health. Further, interpersonal interactions were negatively related with cohesion and perceived individual performance. This pattern may reflect the potential costs of occupying highly central positions in strained social environments. Individuals who interact frequently with many others may become exposed to competing demands, tensions, or conflicting perspectives within the group. In small, confined teams, central actors may even find themselves positioned between emerging factions, a role that can increase psychological strain rather than confer the benefits typically associated with well-connected social networks.

This finding contrasts with research in nonextreme environments, where rich and diverse social networks are often linked to numerous benefits, including increased well-being, social support, and resilience (28, 56, 57). In many settings, more frequent and widespread interactions provide greater opportunities for collaboration, emotional connection, and personal growth. In our context, however, the positive association between interaction frequency and negative outcomes may reflect several important caveats. First, wearable proximity sensors measure the amount, but not the quality of interactions. Proximity does not distinguish between supportive exchanges and conflictual encounters; a heated argument is recorded the same way as a friendly conversation. Given the observed increase in team conflict and psychological strain, it is possible that many of the captured interactions were marked by tension rather than support. Second, individuals experiencing loneliness or mistrust may actively seek out more contact to regulate their distress, but in a small, closed environment with limited social options, their need for connection may remain unmet or be met with frustration, further reinforcing the negative effect (15). The positive association between interactions and loneliness scores was only marginally significant, which may indicate that the increase in interpersonal contacts during the mission was not primarily driven by a need for social connection.

Moreover, evidence indicates that paranoid ideation is accompanied by an expansion of preferred personal space. This is defined as the region immediately surrounding the body that functions as a perceived safety buffer, whose intrusion elicits discomfort or flight responses (58–60) and seems to be linked to heightened physiological stress reactivity as well as to a self-reinforcing stress–paranoia cycle (61). In highly confined settings such as Concordia Station, personal space violations may therefore amplify this dynamic, increasing the risk for social dysfunction and interpersonal conflict.

Finally, because our analyses are correlational, we cannot draw conclusions about causality. It remains unclear whether increased contact exacerbates psychological strain, whether strained individuals engage in more (but less rewarding) interactions, or whether a third factor, such as team structure or role, drives both patterns. These possibilities highlight the need for future research to capture both the quality and intent of social interactions to better understand their psychological and functional implications in ICE environments.

In addition, sensor data revealed the emergence of two subgroups, divided by language and nationality (French, Italian), with within-group interactions increasing over time. This homophilic interaction pattern reflects the tendency for social ties to form more readily among similar others, often summarized as “birds of a feather flock together” (62). At the same time, such patterns may also reflect processes described by social identity theory, which suggests that under conditions of uncertainty or threat, individuals tend to categorize themselves and others into salient in-groups and out-groups and preferentially affiliate with those perceived as similar (63). In our context, shared nationality and language, as well as occupational roles (e.g., researchers vs. technicians) likely provided readily available identity anchors. As cognitive and emotional resources diminished over time, individuals may have increasingly relied on these familiar group boundaries, reinforcing intragroup cohesion at the expense of broader team integration. Others have found similar tendencies toward homophily in other winter-over crews (64, 65). These findings highlight the risk of social fragmentation in multicultural crews and underscore the importance of carefully considering language and cultural diversity in team composition for long-duration missions.

This study uses wearable proximity sensors to assess team interactions during a winter-over Antarctica mission quantitatively. It also represents an investigation in such a setting to examine mild paranoid thinking and perceived social isolation in relation to team functioning. Nevertheless, several limitations should be considered. First, the small sample size limits the generalizability and statistical power of the findings. While repeated measures and within-subject analyses can partly compensate for this, the demanding context of ICE missions inherently restricts sample availability and data completeness. Participant burden and the voluntary nature of participation likely contributed to occasional gaps in sensor data. As reported in the results, field observations by the ESA medical doctor indicated that noncompliance was often deliberate and coincided with episodes of voluntary social withdrawal. However, intentional and unintentional lapses in sensor use are confounded in the available data and cannot be disentangled. This ambiguity may introduce bias into the results, particularly if some missingness reflects technical or incidental factors rather than behavioral withdrawal. Future studies should incorporate structured compliance monitoring procedures to better distinguish between these mechanisms. Moreover, the possibility that voluntary sensor removal may itself constitute a behavioral marker of psychological or social strain warrants systematic investigation.

Second, while Concordia Station offers one of the most realistic analogs for long-duration spaceflight, the unique environmental, cultural, and operational characteristics of this setting may limit the transferability of results to other contexts. Third, although our study could not capture the quality or content of the recorded interactions, we successfully demonstrated that wearable proximity sensors can function reliably in extreme conditions over extended periods, highlighting their potential for future use in space analog and spaceflight research. Finally, the analysis of social interaction remains descriptive but may not account for work schedule-related affordances. Thus, the measured interactions include a mix of job-related and spare-time interactions.

While our sample size was necessarily small, such samples can yield conceptually valuable insights, particularly in extreme environments where large scale studies are difficult to conduct (66). At the same time, we acknowledge that the present study represents a case study of a single winter-over crew, corresponding to an n of 1 at the group level. Therefore, the observed patterns should not be interpreted as inevitable features of group functioning in ICE environments. Instead, team dynamics in other missions may follow different trajectories depending on contextual and interpersonal factors, such as leadership configurations, informal role emergence, or team composition. Supporting this view, research comparing three winter-over crews at the Amundsen Scott South Pole Station found substantial variation across years in the evolution of social network structures as well as psychological and behavioral outcomes (64, 65). Future research should build on these findings by replicating our approach with additional crews, examining potential moderators of social dynamics, and identifying mechanisms that may explain why some teams maintain positive interaction patterns while others experience increasing strain during prolonged isolation.

Taken together, our findings highlight important psychological dynamics that may critically affect the functioning of teams during long-duration space missions. Specifically, mild paranoid symptoms, such as suspiciousness and mistrust, emerged as relevant factors linked to deteriorating team cohesion and increased interpersonal conflict. The association between these symptoms and higher levels of proximity-based interaction suggests that it is not isolation per se, but rather prolonged close physical confinement that may serve as a key precipitating factor in psychosocial strain. Popular culture, such as Stephen King’s The Shining, captures a similar intuition: In prolonged isolation, constant proximity does not necessarily strengthen relationships but can instead amplify tension, mistrust, and psychological strain. While fictional, this reflects real risks observed in ICE environments, where interaction overload may become detrimental rather than protective. These insights underscore the need for targeted investigations into the role of subclinical psychopathological processes in team dynamics in ICE environments. Beyond Antarctic stations, this methodology could be applied in other ICE environments such as submarines, offshore oil and gas platforms, and remote research or military stations.

## Material and Methods

### Study Site.

This study was conducted at Concordia Station, located at Dome C on the Eastern Antarctic Plateau. Concordia is a unique and challenging research facility, jointly operated by the French Polar Institute (Institut Polaire Français Paul-Émile Victor, IPEV) and the Italian Antarctic Programme (Programma Nazionale di Ricerche in Antartide, PNRA). Established in 2004, the station has been continuously inhabited, functioning as a permanent hub for research across diverse fields, including glaciology, atmospheric sciences, astronomy, astrophysics, Earth science, and technology (13).

Situated at an altitude of 3,200 m, inhabitants of Concordia experience reduced oxygen levels and chronic hypobaric hypoxic stress. The environmental conditions are among the harshest on Earth, with a mean winter temperature of −51 °C and extremes reaching as low as −80 °C. Its remote location—950 km from the nearest coast, 1,670 km from the South Pole, and 560 km from the closest neighboring station—further isolates it.

A typical overwintering team at the station consists of 12 to 15 individuals, fulfilling technical and scientific roles. During the Antarctic winter, spanning mid-February to mid-November, Concordia is completely cut off from the outside world, with no possibility of external assistance. Crew members must wear heavy protective suits for any outdoor activities, braving the extreme environment.

### Participants.

Twelve participants, all overwintering crew members at Concordia Station, were included in the study. Written informed consent was obtained from all participants prior to the commencement of the study. The research protocol was approved by the local Ethical Review Board of ETH Zurich (EK 2020-N-144) and received additional ethical approval of ESA.

The final crew composition included participants of Italian and French nationality and one from another ESA member state. The age distribution was as follows: Two participants were aged 25 to 29 y, one was 30 to 34 y old, five were 40 to 44 y old, one was 45 to 49 y old, one was 50 to 54 y old, and two were 55 to 60 y old. Two team members (identified by the letters E and H) left the mission after the first data collection due to medical reasons and were replaced by subjects identified by the letters M and N.

All participants underwent medical and psychological screening before deployment, adhering to the protocols of the ESA, the French Polar Institute (IPEV), and the Italian Antarctic Programme (PNRA). Two participants held a bachelor’s degree (or equivalent), eight had a master’s degree, and two had a PhD.

### Data Collection Procedure.

Data were collected at four time points over the course of the 10-mo mission. Participants wore proximity sensors during the daytime for two consecutive weeks in months 1, 3, 6, and 9. The four measurement points (Months 1, 3, 6, and 9) also correspond to distinct environmental phases at Concordia Station. Specifically, Month 3 coincides with the end of the summer season and the transition into increasing darkness, whereas Month 6 occurs during the peak of the Antarctic winter, characterized by continuous darkness (“polar night”). Psychometric self-assessments measuring feelings of isolation, paranoia, team cohesion, conflict, and perceived individual performance, focusing on the previous 2 wk, were administered at the end of each data collection period. The full survey instrument is provided in *SI Appendix*.

Participants were instructed to wear the sensors consistently throughout the data collection periods, except during nighttime. Participants were allowed to interrupt data collection at any time by removing the battery from the sensors if they wished to do so. Stationary sensors were installed on the walls of selected spaces within the station, serving as a proxy for the location where interactions occurred.

### Questionnaires.

#### Loneliness.

Loneliness is a subjective emotional experience that arises from the perceived gap between desired and actual social connections. It reflects feelings of social isolation, even when an individual is not physically isolated. Loneliness was assessed using the UCLA Loneliness Scale (67), a 20-item instrument designed to measure participants’ feelings of social disconnection and dissatisfaction with their interpersonal relationships. The scale evaluates both the frequency and intensity of loneliness. A sample item includes: “How often do you feel that you lack companionship?.” Response options ranged from 1 (never) to 4 (always). Scores are summed together, with a maximum possible score of 80 (representing the maximum degree of loneliness) and a minimum score of 20. Internal consistency across measurement occasions was α = 0.84-0.94.

#### Paranoid thinking.

Paranoid thinking was assessed using the Green et al. Paranoid Thoughts Scale (GPTS; 68). This 32-item scale measures two key dimensions (16 items each): ideas of reference (the belief that others are observing or talking about the individual) and persecutory ideation (the belief that others intend harm). A sample item for ideas of reference includes: “I spent time thinking about crew members gossiping about me.” A sample item for ideas of persecution includes: “I was sure certain people did things in order to annoy me.” The scale has been psychometrically evaluated for use in both clinical and nonclinical samples. Response options range from 1 (not at all) to 5 (totally). Scores are summed together, with a maximum possible score of 80 and a minimum score of 16 for each dimension. Internal consistency was high for both subscales across measurement occasions (ideas of reference: α = 0.85-0.96; persecutory ideation: α = 0.95-0.96).

#### Team cohesion.

Team cohesion refers to the degree to which team members are united in pursuing shared goals and maintaining positive interpersonal relationships. It encompasses both the task-focused and social aspects of group unity, as well as the sense of belonging within a team. Team cohesion was assessed using a 6-item scale developed by Mathieu (69). A sample item includes: “During the last 2 wk, there was a feeling of unity and cohesion in my team.” Response options ranged from 1 (strongly disagree) to 5 (strongly agree). Internal consistency across measurement occasions was α = 0.93-0.98.

#### Team conflict.

Conflict is the perception of incompatibilities or disagreements among team members that arise during collaboration. Team conflict was assessed with six items by Jehn and Mannix (70), covering task conflict (disagreements about work-related tasks, goals) and relationship conflict (disagreements rooted in personal tensions, emotions, or interpersonal issues), with three items per subscale. For the main analyses, these items were averaged to form an overall team conflict index reflecting general conflict within the team; subscale level information is provided in *SI Appendix*, Fig. S1 and Table S1. A sample item includes: “In the last 2 wk, how much conflict of ideas was there in your crew?.” Response options ranged from 1 (none) to 5 (a great deal). Internal consistency across measurement occasions was α = 0.86-0.93.

#### Individual performance.

Individual performance was assessed using a 3-item measure based on Aubé (71). The items focus on reaching individual goals and the quality of one’s work. A sample item includes: “During the last 2 wk, I attained my assigned performance goals.” Response options ranged from 1 (strongly disagree) to 5 (strongly agree). Internal consistency across measurement occasions was α = 0.91-0.96.

### Proximity Sensors.

We used wearable proximity sensors developed by the SocioPatterns collaboration (http://www.sociopatterns.org; 24). These devices detect close-range, face-to-face proximity interactions within approximately 1 to 1.5 m at high temporal resolution. In contrast to survey-based network measures, which capture individuals’ subjective perceptions of social relationships, sensor-based approaches provide objective behavioral data on patterns of physical copresence and interaction opportunities between individuals. The technology has been extensively deployed across diverse real-world settings, including schools (27, 72), hospital wards (73), and households in sub-Saharan Africa (26, 27), among others. In addition to broad empirical use across contexts, the sensors have been validated against self-reported contact diaries (74). This study shows substantial agreement between the two approaches in identifying recurrent interaction partners and broader network structures such as communities. At the same time, each method captures different aspects of social interaction: Self-reports provide insight into the perceived quality and meaning of relationships, whereas sensor-based measurements offer a fine-grained and temporally precise record of face-to-face proximity events, avoiding recall biases that can affect retrospective reports (74). We chose wearable proximity sensors because repeated self-report assessments of social interactions can be burdensome and prone to recall bias, particularly in teams operating under demanding conditions. Sensors allow continuous, unobtrusive measurement of behavioral interaction patterns and their evolution over time.

The sensors are worn on the chest and detect face-to-face proximity through the exchange of low-power radio packets in the Industrial, Scientific, and Medical band. When two devices are within close range, they exchange data packets and record a timestamp along with the received signal strength indicator, which reflects signal attenuation and serves as a proxy for distance. Proximity data are stored in the device’s internal memory and subsequently downloaded for analysis. During postprocessing, the chosen definition of contact is applied to suitably filtered and smoothed data. For this study, a contact is defined as a sustained proximity interaction at less than 1 to 1.5 m, with a temporal resolution of 10 s. Each device has a unique hardware identifier that is linked to a specific participant and their corresponding questionnaire data. For our data collection, we mounted the sensors on lanyards worn around the neck and enclosed in protective casings. Preliminary field tests showed that plastic sleeves promoted static charge buildup in the low-humidity conditions at Concordia Station; sensors were therefore housed in paper envelopes to limit static accumulation and reduce interference with functioning.

### Statistical Methods.

For the questionnaire data, we initially used an ANOVA with repeated measures to investigate changes in the variables over time. To provide a more robust analysis of the longitudinal data and to make use of all available observations, we also estimated latent growth models. In addition, we implemented a bootstrap procedure based on Monte Carlo resampling (2,000 resamples) to obtain robust CI for the model parameters that do not rely on asymptotic assumptions.

For the network analysis, we treated each deployment as an independent measurement and aggregated all the contacts that occurred during that span of time. Given the small size of the community under study and the specific work and living conditions, the social network is fully connected, i.e., all individuals interact with one another. Consequently, the information on interaction patterns lies in the timing and duration of interactions. A straightforward way to summarize high-resolution proximity data over a given time interval is to create a static weighted graph describing the interactions over that interval. In this representation, the weight of an edge (interaction) connecting two nodes (subjects) is defined as the total time those nodes spent in proximity during the chosen time interval. In the following, we focus on two main network metrics for each node: the strength centrality^*^ of a node (i.e., the sum of the weights of all edges incident on that node) and the Gini coefficient of the weight distribution across all edges for a given node (75). High Gini coefficients indicate that interaction time is dominated by a single edge, i.e., that an individual tends to interact mainly with the same person; conversely, low Gini coefficients indicate that the edge weights are more homogeneously distributed, and interactions are less selective.

We investigated the correlation between pairs of psychological or network attributes by looking at i) within-subject and across-time correlations; ii) across-subject correlations. Positive within-subject correlation between two measures x, y (e.g., loneliness and strength) implies that when a person reported a higher x value, they typically reported high y values as well. Positive across-subject correlation between x and y means that individuals with a higher x typically also have large y values, compared to others. We write

$$x_{p,i}(t)={\overset{-}{x}}_{p,i}+\delta_{p,i}(t),$$

where $x_{p,i}\left(t\right)$ is the measure *i* (strength, entropy, loneliness, conflict, etc.) of person *p* during deployment *t*; ${\overset{-}{x}}_{p,i}$ is the average of the *i-*th measurement of the *p*-th person over the deployments and $\delta_{p,i}\left(t\right)$ denotes the variation from the mean. In other words, the values ${\overset{-}{x}}_{p,i}$ allow for making a comparison between individuals, while the variations $\delta_{p,i}\left(t\right)$ allow for comparing every individual’s scores across time. This decomposition corresponds to person-mean centering of the variables, such that within-subject correlations are based on deviations from individual means. The correlations are quantified using the Spearman coefficient.

## Supplementary Material

Appendix 01 (PDF)

## Data Availability

Anonymized CSV data have been deposited in OSF (https://osf.io/v2n74/overview?view_only=d8303c994e1d455a9a8ed015008d2db6) (76).

## Footnotes

^*^Strength centrality is a weighted notion of node centrality in a network. It is defined as the sum of the weights of all edges incident on a node. In temporal interaction networks, it is expressed in units of time, and it can exceed the total duration of the observation window (e.g., three people interacting for 20 min each accumulate 40 min). As such, daily strength is not bounded by 24 h.
