# Supplementary material for: Social interactions in isolated, confined, and extreme environments: A study of Antarctic winter teams using wearable sensors
Source: Proc Natl Acad Sci U S A. 2026 May 26;123(22):e2533420123. doi: 10.1073/pnas.2533420123 (PMC13229265; doi:10.1073/pnas.2533420123)
Supplement: Supplementary file 1 — Appendix 01 (PDF) [file pnas.2533420123.sapp.pdf]

PNAS

Supporting Information for
Social Interactions in Isolated, Confined, and Extreme Environments: A Study of Antarctic Winter Teams Using Wearable Sensors

Andrea Cantisani[1,2,a], Jan B. Schmutz* [3,a], Pedro Marques-Quinteiro[4, 5], Lorenzo Dall'Amico[6], Ciro Cattuto[6], Mirko Antino[7], Walter J. Eppich[8], Katharina Stegmayer[1], Sebastian Walther[1, 9]

[1] University Hospital of Psychiatry and Psychotherapy, University of Bern, Bern, Switzerland

[2] Sanatorium Kilchberg, Private Clinic for Psychiatry and Psychotherapy, Kilchberg, Switzerland

[3] Department of Psychology, University of Zurich, Switzerland

[4] Intrepid Lab, ECEO, Lusófona University, Lisbon, Portugal

[5] CETRAD, Centre for Transdisciplinary Development Studies, UTAD, Vila Real, Portugal

[6] ISI Foundation, Torino, Italy

[7] Universidad Complutense de Madrid

[8] Faculty of Medicine, Dentistry, and Health Sciences, University of Melbourne, Australia

[9] Department of Psychiatry, Psychosomatics, and Psychotherapy, Center of Mental Health, University Hospital of Würzburg, Würzburg, Germany

[a]shared first authorship, A.C. and J.B.S. contributed equally to this work.

*Jan B. Schmutz
University of Zurich
Department of Psychology
Binzmühlestr. 14
8050 Zurich.

Email: jan.schmutz@psychologie.uzh.ch

This PDF file includes:

Figure S1
Table S1
Supporting Text

Figures

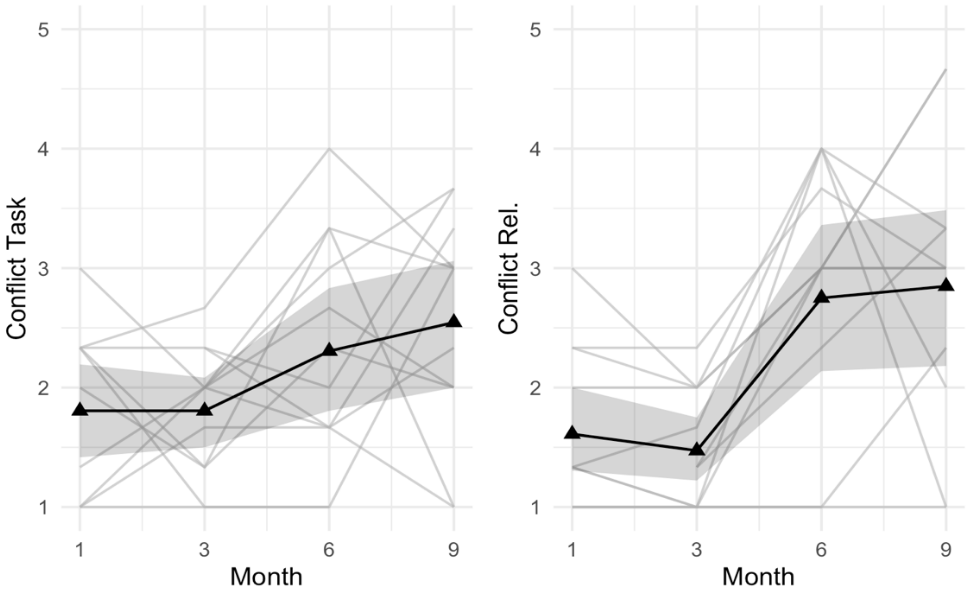

Fig. S1. Changes in task conflict and relationship conflict across the four measurement time points (months 1, 3, 6, and 9)

Tables

Table S1. Means, standard deviations, and correlations among study variables across four measurement time points.

		M	*SD*	1	2	3	4	5	6	7	8	9	10	11	12	13	14	15	16	17	18	19	20	21	22	23	24	25	26	27	28	29	30	31
1	Loneliness (t1)	41.00	5.33	–																														
2	Loneliness (t2)	41.91	6.91	0.18	--																													
3	Loneliness (t3)	44.08	7.87	0.37	0.78**	--																												
4	Loneliness (t4)	48.58	10.81	0.49	0.75**	0.88**	--																											
5	Paranoia Social Reference (t1)	16.33	0.65	-0.50	0.15	0.19	-0.13	--																										
6	Paranoia Social Reference (t2)	16.08	0.29	-0.41	0.48	0.56	0.33	0.67*	--																									
7	Paranoia Social Reference (t3)	20.75	9.77	-0.51	0.30	0.39	0.09	0.92**	0.88**	--																								
8	Paranoia Social Reference (t4)	19.00	13.09	-0.15	0.64*	0.53	0.31	0.69*	0.70*	0.78**	--																							
9	Paranoia Persecution (t1)	19.00	3.67	-0.41	0.46	0.52	0.18	0.91**	0.82**	0.96**	0.81**	--																						
10	Paranoia Persecution (t2)	18.50	4.60	-0.32	0.72*	0.69*	0.46	0.68*	0.92**	0.84**	0.81**	0.88**	--																					
11	Paranoia Persecution (t3)	22.42	10.39	-0.14	0.59	0.64*	0.37	0.62	0.90**	0.87**	0.81**	0.86**	0.92**	--																				
12	Paranoia Persecution (t4)	21.42	9.57	-0.18	0.76**	0.72**	0.54	0.60	0.87**	0.79**	0.85**	0.85**	0.95**	0.91**	--																			
13	Cohesion (t1)	4.13	0.65	-0.55	-0.31	-0.56	-0.61	0.18	0.01	-0.01	-0.09	0.06	-0.03	-0.09	-0.08	--																		
14	Cohesion (t2)	4.24	0.60	-0.24	-0.33	-0.09	-0.12	0.18	-0.12	0.02	-0.13	-0.05	-0.19	-0.24	-0.21	-0.04	--																	
15	Cohesion (t3)	3.43	0.92	-0.45	0.10	-0.11	-0.08	0.09	0.08	0.00	0.01	0.01	0.12	-0.09	0.08	0.51	0.56	--																
16	Cohesion (t4)	2.93	1.07	-0.48	-0.18	-0.20	-0.48	0.38	0.22	0.38	0.14	0.30	0.19	0.25	0.10	0.57	0.48	0.53	--															
17	Conflict (t1)	1.71	0.62	0.26	0.37	0.68*	0.41	0.26	0.49	0.51	0.40	0.55	0.56	0.68*	0.62	-0.31	-0.28	-0.29	0.07	--														
18	Conflict (t2)	1.64	0.47	0.33	0.62*	0.61*	0.35	0.31	0.47	0.48	0.73**	0.61	0.62*	0.74**	0.69*	-0.19	-0.55	-0.43	-0.07	0.72*	--													
19	Conflict (t3)	2.53	0.97	0.08	0.26	0.32	0.16	0.39	0.31	0.47	0.37	0.53	0.42	0.52	0.39	-0.37	-0.60*	-0.68*	-0.26	0.61	0.63*	--												
20	Conflict (t4)	2.61	1.04	0.10	0.27	0.12	0.07	0.15	0.12	0.11	0.48	0.26	0.26	0.19	0.33	0.36	-0.40	0.04	-0.14	0.25	0.51	0.14	--											
21	Task Conflict (t1)	1.81	0.70	0.24	-0.03	0.43	0.22	0.41	0.23	0.36	0.30	0.22	0.23	0.30	0.28	-0.29	-0.11	-0.31	0.04	.92**	0.42	0.51	0.09	--										
22	Task Conflict (t2)	1.81	0.54	0.22	.65*	.55*	0.35	0.53	.60*	.63*	.66*	0.25	.50*	0.37	.52*	-0.12	-.67**	-0.40	-0.24	0.54	.90**	0.49	.53*	0.21	--									
23	Task Conflict (t3)	2.31	0.97	0.07	0.37	.60*	0.40	.64*	.62*	.66**	.53*	0.45	.55*	.59*	0.50	-0.47	-0.45	-.56*	-0.21	.71*	.62*	.89**	0.05	.56*	.51*	--								
24	Task Conflict (t4)	2.55	0.96	0.13	0.28	0.20	0.14	0.20	0.29	0.19	0.32	0.03	0.16	0.06	0.20	.59*	-0.27	0.35	0.06	0.31	0.43	-0.06	.95**	0.16	0.43	-0.01	--							
25	Relationship Conflict (t1)	1.61	0.66	0.25	.69*	.80**	0.53	.60*	.78**	.89**	.83**	0.26	.67*	.631*	.76**	-0.28	-0.39	-0.22	0.08	.91**	.90**	.60*	0.37	.67**	.78**	.72**	0.42	--						
26	Relationship Conflict (t2)	1.47	0.50	0.38	0.45	.54*	0.27	.58*	0.49	.69**	.57*	0.32	0.33	0.49	.57*	-0.23	-0.30	-0.36	0.13	.78**	.88**	.64*	0.38	.56*	.59*	.59*	0.33	.86**	--					
27	Relationship Conflict (t3)	2.75	1.17	0.08	0.11	0.04	-0.06	0.38	0.18	0.31	0.21	0.30	0.07	0.30	0.30	-0.25	-.63*	-.66**	-0.26	0.46	.54*	.93**	0.19	0.40	0.39	.65*	-0.09	0.42	.58*	--				
28	Relationship Conflict (t4)	2.85	1.22	-0.06	0.07	-0.06	-0.11	0.23	0.16	0.09	0.26	0.16	0.04	0.08	0.25	.68*	-0.35	0.14	0.04	0.22	0.43	0.04	.97**	0.15	0.46	-0.13	.84**	0.26	0.30	0.17	--			
29	Individual effectiveness (t1)	4.50	0.64	-0.67*	-0.50	-0.60	-0.46	0.22	-0.21	-0.16	-0.45	-0.05	-0.33	-0.50	-0.42	0.38	0.27	0.13	0.06	-0.60*	-0.70*	-0.28	-0.47	-0.39	-0.51	-0.34	-0.61*	-.71**	-.78**	-0.20	-0.26	--		
30	Individual effectiveness (t2)	4.47	0.50	-0.17	-0.14	-0.05	0.01	0.04	-0.30	-0.20	-0.14	-0.18	-0.22	-0.42	-0.25	0.07	0.85**	0.63*	0.28	-0.41	-0.52	-0.65*	-0.09	-0.25	-.56*	-.51*	0.05	-0.49	-0.37	-.66*	-0.03	0.30	--	
31	Individual effectiveness (t3)	4.33	0.64	-0.57	-0.25	-0.47	-0.50	0.18	-0.17	-0.09	-0.05	-0.08	-0.17	-0.34	-0.22	0.58	0.36	0.41	0.41	-0.56	-0.34	-0.55	0.05	-0.50	-0.15	-.57*	0.01	-0.52	-0.48	-0.43	0.26	0.67*	0.51	--
32	Individual effectiveness (t4)	3.75	1.15	-0.20	-0.09	-0.31	-0.37	-0.07	-0.21	-0.18	0.02	-0.19	-0.15	-0.22	-0.17	0.62	0.21	0.34	0.48	-0.40	-0.07	-0.54	0.20	-0.44	0.05	-.55*	0.23	-0.28	-0.18	-0.45	0.38	0.28	0.40	0.86**

Note. *N* ranges between 9 and 12 due to missing data; * Correlation is significant at the 0.05 level (2-tailed); ** Correlation is significant at the 0.01 level (2-tailed).

Supporting Text

Supporting Text S1. Survey Instrument

Project HD-ICE
Social Dynamics in Antarctica

Survey End of 1st Period

ENGLISH

1

Thank you for supporting our project about teamwork in Antarctica.

This is the end of the first data collection period with the sensory badges. We would like to ask you a few questions. There is no right or wrong answer. The data will be analysed anonymously using your personal badge ID only. If you have any questions the ESA research MD can provide you more information.

What is your personal badge ID (number on the badge*): ____________________

*ESA research MD will help you find the number

The following questions focus on how you have worked on your team **over the past two weeks.**

Team climate

In the last two weeks…

	Totally disagree	Disagree	Neither agree nor disagree	Agree	Totally agree
There was a feeling of unity and cohesion in my team	☐	☐	☐	☐	☐
There was a strong feeling of belongingness among my team members	☐	☐	☐	☐	☐
Members of my team felt close to each other	☐	☐	☐	☐	☐
Members of my team shared a focus on our work	☐	☐	☐	☐	☐
My team concentrated on getting things done	☐	☐	☐	☐	☐
My team members pulled together to accomplish work	☐	☐	☐	☐	☐

Conflict in your team

In the last two weeks…

	None	Rarely	Some-times	Often	A great deal
How much conflict of ideas was there in your workgroup?	☐	☐	☐	☐	☐
How frequently did you have disagreements within your work group about the task of the project you are working on?	☐	☐	☐	☐	☐
How often did people in your work group have conflicting opinions about the project you are working on?	☐	☐	☐	☐	☐
How much relationship tension was there in your work group?	☐	☐	☐	☐	☐
How often did people get angry while working in your work group?	☐	☐	☐	☐	☐
How much emotional conflict was there in your work group?	☐	☐	☐	☐	☐

Productivity

Think back to the last two week. To what extent do you agree with the following statements about the productivity of your team and you as an individual?

In the last two weeks, me as an individual…

	Totally disagree	Disagree	Neither agree nor disagree	Agree	Totally agree
I reached my assigned performance goals	☐	☐	☐	☐	☐
I produced quality work	☐	☐	☐	☐	☐
I was productive	☐	☐	☐	☐	☐

The following questions now focus on your individual experience on the station. The statements refer to thoughts or feelings you may have had in **the last two weeks.** Rate the frequency of these thoughts or feelings on a scale of 1 (never) to 4 (often).

In the last two weeks…

	never	rarely	some-times	often
I felt in tune with the people around me	☐	☐	☐	☐
I lacked companionship	☐	☐	☐	☐
There was no one I can turn to	☐	☐	☐	☐
I did not feel alone	☐	☐	☐	☐
I felt part of a group of friends	☐	☐	☐	☐
I had a lot in common with the people around me	☐	☐	☐	☐
I was no longer close to anyone	☐	☐	☐	☐
My interests and ideas were not shared by those around me	☐	☐	☐	☐
I am an outgoing person	☐	☐	☐	☐
There were people I feel close to	☐	☐	☐	☐
I felt left out	☐	☐	☐	☐
My social relationships were superficial	☐	☐	☐	☐
No one really knew me well	☐	☐	☐	☐
I felt isolated from others	☐	☐	☐	☐
I could find companionship when I want it	☐	☐	☐	☐
There were people who really understand me	☐	☐	☐	☐
I was unhappy being so withdrawn	☐	☐	☐	☐
People were around me but not with me	☐	☐	☐	☐
There were people I could talk to	☐	☐	☐	☐
There were people I can turn to	☐	☐	☐	☐

Please read each of the statements carefully. They refer to thoughts and feelings you may have had about others over **the last two weeks**. Think about the last two weeks and indicate the extent of these feelings from 1 (Not at all) to 5 (Totally).

In the last two weeks…

	Not at all	Rarely	Some-times	Often	Totally
I spent time thinking about friends gossiping about me	☐	☐	☐	☐	☐
I often heard people referring to me	☐	☐	☐	☐	☐
I have been upset by friends and colleagues judging me critically	☐	☐	☐	☐	☐
People definitely laughed at me behind my back	☐	☐	☐	☐	☐
I have been thinking a lot about people avoiding me	☐	☐	☐	☐	☐
People have been dropping hints for me	☐	☐	☐	☐	☐
I believed that certain people were not what they seemed	☐	☐	☐	☐	☐
People talking about me behind my back upset me	☐	☐	☐	☐	☐
I was convinced that people were singling me out	☐	☐	☐	☐	☐
I was certain that people have followed me	☐	☐	☐	☐	☐
Certain people were hostile towards me personally	☐	☐	☐	☐	☐
People have been checking up on me	☐	☐	☐	☐	☐
I was stressed out by people watching me	☐	☐	☐	☐	☐
I was frustrated by people laughing at me	☐	☐	☐	☐	☐
I was worried by people's undue interest in me	☐	☐	☐	☐	☐
It was hard to stop thinking about people talking about me behind my back	☐	☐	☐	☐	☐

In the last two weeks...

	Not at all	Rarely	Some-times	Often	Totally
Certain individuals have had it in for me	☐	☐	☐	☐	☐
I have definitely been persecuted	☐	☐	☐	☐	☐
People have intended me harm	☐	☐	☐	☐	☐
People wanted me to feel threatened, so they stared at me	☐	☐	☐	☐	☐
I was sure certain people did things in order to annoy me	☐	☐	☐	☐	☐
I was convinced there was a conspiracy against me	☐	☐	☐	☐	☐
I was sure someone wanted to hurt me	☐	☐	☐	☐	☐
I was distressed by people wanting to harm me in some way	☐	☐	☐	☐	☐
I was preoccupied with thoughts of people trying to upset me deliberately	☐	☐	☐	☐	☐
I couldn't stop thinking about people wanting to confuse me	☐	☐	☐	☐	☐
I was distressed by being persecuted	☐	☐	☐	☐	☐
I was annoyed because others wanted to deliberately upset me	☐	☐	☐	☐	☐
The thought that people were persecuting me played on my mind	☐	☐	☐	☐	☐
It was difficult to stop thinking about people wanting to make me feel bad	☐	☐	☐	☐	☐
People have been hostile towards me on purpose	☐	☐	☐	☐	☐
I was angry that someone wanted to hurt me	☐	☐	☐	☐	☐

Did something significant happen in the last two weeks related to your team and/or your project?
